# Androgen modulation of social decision-making mechanisms in the brain: an integrative and embodied perspective

**DOI:** 10.3389/fnins.2014.00209

**Published:** 2014-07-22

**Authors:** Gonçalo A. Oliveira, Rui F. Oliveira

**Affiliations:** ^1^Unidade de Investigação em Eco-Etologia, ISPA – Instituto UniversitárioLisboa, Portugal; ^2^Integrative Behavioural Biology Lab, Instituto Gulbenkian de CiênciaOeiras, Portugal; ^3^Champalimaud Neuroscience Program, Champalimaud Center for the UnknownLisboa, Portugal

**Keywords:** androgens, testosterone, ultimate causes, proximate causes, embodiment, challenge hypothesis

## Abstract

Apart from their role in reproduction androgens also respond to social challenges and this response has been seen as a way to regulate the expression of behavior according to the perceived social environment (Challenge hypothesis, Wingfield et al., 1990). This hypothesis implies that social decision-making mechanisms localized in the central nervous system (CNS) are open to the influence of peripheral hormones that ultimately are under the control of the CNS through the hypothalamic-pituitary-gonadal axis. Therefore, two puzzling questions emerge at two different levels of biological analysis: (1) Why does the brain, which perceives the social environment and regulates androgen production in the gonad, need feedback information from the gonad to adjust its social decision-making processes? (2) How does the brain regulate gonadal androgen responses to social challenges and how do these feedback into the brain? In this paper, we will address these two questions using the integrative approach proposed by Niko Tinbergen, who proposed that a full understanding of behavior requires its analysis at both proximate (physiology, ontogeny) and ultimate (ecology, evolution) levels.

## Introduction

In his classical paper “On aims and methods of Ethology,” Niko Tinbergen (1963) identified proximate causation, survival value, ontogeny and evolution as the four major questions in the study of behavior. Although these four questions can be interpreted as culminating into the proximate-ultimate dichotomy of biological causation proposed by Mayr (1961), Tinbergen's formulation clearly distinguishes cause from function and calls not the separateness of his questions, but rather for their integration when investigating a particular phenotype. Only such an integrative approach would allow a truly comprehensive understanding of the behavior in question. Indeed, on one hand knowledge of the proximate mechanisms underlying a given behavior is crucial to understanding its costs, limits and evolutionary consequences, therefore highlighting the fact that proximate mechanisms contribute to the dynamics of selection. On the other hand, knowledge on the ecological function and evolution of a given behavior will clarify how the proximate mechanisms underlying it evolved. Thus, reciprocal causation analysis of biological phenomena (i.e., considering the interaction between immediate factors and evolutionary explanations) can be a more useful approach than the traditional proximate-ultimate dichotomy (e.g., Laland et al., 2013).

Following Tinbergen's footsteps, here we aim to integrate the study of function with the study of proximate mechanisms of the social modulation of androgens. For this purpose we will start by reviewing the current hypothesis for the social modulation of androgen levels, we will then address its proximate and ultimate mechanisms, and we will finish by integrating both levels of analysis in addressing the ultimate question of why are social decision-making mechanisms in the brain open to modulation by peripheral hormones. The term function will be used here in reference to the current utility of a character, as it makes no assumptions about the processes from which function emerged and emphasizes that current and original function may not match (Bateson and Laland, 2013).

## Reciprocal models of androgen-social behavior interactions

Over the last decades, accumulated evidence has revealed a reciprocal relationship between androgen levels and the social environment. As a result, androgens are no longer seen exclusively as sex steroids involved in reproduction. Early models for the interaction between hormones and behavior (Leshner, 1975, 1979; Mazur, 1976), already presented the core ideas that would be further developed in subsequent formal explanations, namely that androgen levels influence the behavioral response to social stimuli and that changes in androgens can be elicited by the social environment, thus creating a reciprocal interaction between androgens and behavior [i.e., biosocial model, (Mazur, 1985); challenge hypothesis, (Wingfield et al., 1990)].

The reciprocal model of androgens and social behavior has been formalized in two different hypotheses, each presenting different theoretical constraints and generating its own predictions. The biosocial model, initially proposed by Mazur (Mazur, 1985; Mazur and Booth, 1998), establishes a dynamic and mutual reinforcing relationship between androgens and social dominance. According to this model, androgens promote status seeking behaviors, and the achievement of higher status through dominance contests feeds back on the individuals' androgen levels, according to the individual's new position in the social hierarchy. Therefore, the biosocial model predicts that dominant individuals should have higher baseline levels of androgens than subordinates and while it is expected that winning an agonistic interaction results in increased androgen levels, establishing a positive feedback loop between status and androgens, losing such an interaction should result in decreased androgens and an inhibition of the individuals' engagement in further dominance contests (Mazur and Booth, 1998).

**Figure 1:**
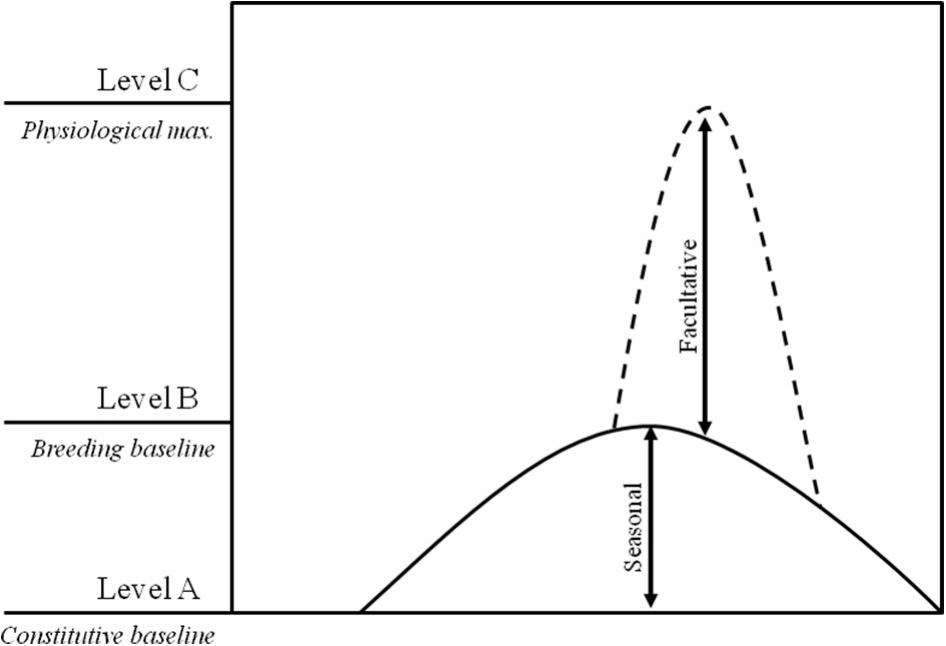
**Representation of the androgen changes proposed by the challenge hypothesis: **(A)** constitutive androgen levels; **(B)** breeding baseline levels needed for successful reproduction; and **(C)** maximum physiological levels**.

While the biosocial model focused essentially on androgens and social dominance, Wingfield and co-workers proposed the “challenge hypothesis” with the goal of providing an explanation for the interspecific seasonal variation of androgen levels, linking fluctuations in androgen levels with its functions in reproductive and aggressive contexts (Wingfield et al., 1990). The “challenge hypothesis” (Figure 1) predicts that androgen levels increase from a non-breeding constitutive baseline (level A) to breeding season levels (level B) to allow for the expression of secondary sex characters and reproductive behaviors; short term further increases in androgen levels up to a maximum physiological level (level C) may occur in response to agonistic encounters (e.g., territorial intrusions). Recent revisions of the “challenge hypothesis” have shown that B to C increases do not reflect the effect of social challenges and in fact, across species, no correlation was found between seasonal androgen responsiveness and the androgen response to an experimental territorial challenge (Goymann et al., 2007). These two time scales of the androgen response to the social environment are expected to rely on different mechanisms (e.g., non-genomic and genomic steroid action: Baker, 2003; Balthazart et al., 2006), and thus should be seen as separate phenomena. For example, while the dynamic reciprocal changes of the biosocial model and of the acute response to a territorial intrusion in the “challenge hypothesis,” are acute and short-lived and therefore are expected to rely on either non-genomic or on transient changes in gene expression, seasonal changes in androgen responses are gradual and long-lasting, and therefore are expected to rely on genomic and epigenetic mechanisms.

## Mechanisms of androgen response to social challenges

Most androgen production results from the activation of the hypothalamic-pituitary-gonadal (HPG) axis in which a sequential pulsatile hormonal cascade targets the Leydig cells in male gonads, to elicit testosterone (T) production and its release into circulation (Gleason et al., 2009). Androgens can also be produced in the brain *de novo* from cholesterol and can be converted into other hormones (Schmidt et al., 2008) and both processes can be modulated by social context (e.g., Pradhan et al., 2010; Cornil et al., 2012). In fact, studies in several taxa (fish, birds, mammals) suggest that the effects of androgens on agonistic behavior is mediated by their rapid aromatization into estrogens in the brain (Soma et al., 2003; Trainor et al., 2006; Charlier et al., 2011; Huffman et al., 2013). Additionally, tissue sensitivity to androgens can also be socially modulated through rapid changes in androgen receptor expression (Burmeister et al., 2007; Fuxjager et al., 2010).

The adjustment of androgen levels according to the social environment requires mechanisms that can translate and integrate multi-modal social information relevant to the organism and modulate neuroendocrine activity responsible for the production of androgens. Cichlid fish have been a very successfully model in this respect. Experiments with cichlid fish have shown how changes in social status can induce rapid changes in HPG axis activity leading to changes in circulating androgens (for comprehensive reviews see Oliveira, 2009; Maruska and Fernald, 2013). When opportunities to ascend in social status arise subordinates can rapidly exhibit the traits of dominant fish (e.g., coloration and aggressive behavior), and sequentially increase the expression of GnRH1 in the preoptic area, pituitary gonadotropins and androgen levels (Maruska et al., 2013). Conversely, dominant males experiencing a decrease in social status present a reduced expression of GnRH1 and pituitary gonadotropins, and a decrease of androgen levels (Maruska et al., 2013). Furthermore, the social information signaling social opportunity seems to be conveyed by changes in the expression of the immediate early gene *egr-1* in high density GnRH1 neuron areas of the anterior preoptic area, indicating that *egr-1* is interfacing social information with the activity of the HPG (Burmeister et al., 2005). Interestingly, experiments where the use of mirror elicited fights allowed for decoupling the effects of expressing aggressive behavior from those of assessing the fight outcome indicate that the androgen response to social status depends on the fish appraisal of the interaction outcome (Oliveira et al., 2005; see also Oliveira and Canário, 2011 for a debate on contradictory results on this topic). Evidence also exists in support of appraisal as a modulator of the androgen response to social contests in birds (e.g., Japanese quail; Hirschenhauser et al., 2008) and in humans (for a recent review see Oliveira and Oliveira, 2014). For example, T changes in female competitors that lost a face to face contest are moderated by the subjective evaluation of the outcome as a threat and the perception of opponent familiarity, with the highest increases of T appearing in situations of perceived high threat with unfamiliar opponents (Oliveira et al., 2013).

## The function of androgen response to social challenges

The fact that androgen levels change in response to the perceived outcome of an interaction, and not merely by experiencing an agonistic interaction raises the hypothesis that socially driven changes in androgen levels will not directly affect the current interaction, for which the outcome has already been established, but should rather modulate behavioral expression in subsequent social interactions (Oliveira, 2009). Interestingly, Leshner's (1975) proposal for the reciprocal model had already hinted that the hormone response should modify future behavior when the individuals are facing a similar challenge, and both the biosocial model and the challenge hypothesis have also implicitly assumed that the adaptive function of the social modulation of androgen levels is to fine tune the expression of androgen-dependent behavior according to the perceived social environment.

More recently, this view has been formalized as the Winning hypothesis (Oyegbile and Marler, 2005) according to which changes in the probability of winning future interactions driven by the success in previous ones (i.e., winner/loser effect, Hsu et al., 2006), could be mediated by post-contest transient changes in androgen levels. This hypothesis is currently supported by several lines of evidence. In cichlid fish winner effects can be blocked (i.e., reduction of the winning probability of previous winners from ca. 90% back to chance levels) by the exogeneous administration of the anti-androgen cyproterone acetate to the winners of the first interaction between the agonistic encounters (Oliveira et al., 2009). In California mice (*Peromyscus californicus*), in the emergence of the winner effect during successive social interactions is paralleled by increased levels of androgens after cumulative winning experience (Oyegbile and Marler, 2005). Furthermore, unlike the California mice, the white-footed mouse (*Peromyscus leucopus*) does not form a winner effect or respond to a contest with increased T, but a robust winner effect can be induced in this species via a post-contest administration of T (Fuxjager et al., 2011). As it has been previously suggested, it is possible that these effects could result from the aromatization of T in the brain (e.g., Trainor et al., 2006). In humans, although to the best of our knowledge no formal tests have been conducted, it is known that increased androgen levels after a competition predict the willingness to engage in further contests, even after losing the first interaction (Mehta and Josephs, 2006; Carré and McCormick, 2008).

One assumption of the Winning hypothesis is that socially driven changes in androgen levels modulate the expression of variables relevant for success in subsequent social contests. Given the time frame of this response these variables are expected to be of the cognitive (i.e., information-processing) domain. Most of the evidence for the effects of androgens on cognitive variables comes from research using paradigms that involve the administration of exogenous T to animals and humans (for a review see Bos et al., 2012), which have been shown to reduce vigilance (Van Honk et al., 2005), startle reflex (Hermans et al., 2006) and threat detection in human females (Van Honk and Schutter, 2007), and to reduce fear in other animals (Frye and Seliga, 2001; Aikey et al., 2002). Furthermore, in women T also reduces trust (Bos et al., 2010), increases risk-taking accompanied by increased sensitivity to rewards and reduced sensitivity to punishment (Van Honk et al., 2004), and also facilitates resource acquisition and high status via cooperation (Eisenegger et al., 2010). Thus, the available data indeed suggests that increased levels of T induce competitive cognitive traits that are beneficial in competitive settings. However, these results should be interpreted with caution since most manipulations used dosages way above the androgens levels observed in response to social challenges. Another issue to consider is that in some species of birds the levels of high aggression toward the intruder are accompanied by lowering T levels (Goymann, 2009). The ecological and adaptive function of this decrement of androgens is still unknown and currently stands outside the predictions of the challenge hypothesis and the biosocial model.

## Modulation of social decision-making mechanisms in the brain by peripheral hormones

If one considers that the social environment is sensed by the brain and that the androgen response to it is a top-down process conveyed by the HPG axis, then, under classical models of cognition, the involvement of peripheral androgens in the modulation of a central decision-making process seems redundant, since the decision-making mechanism already has the relevant information on the social environment and could provide a faster and more economic response *per se*. However, if one shifts perspective toward embodiment as an essential component of cognition, then neuroendocrine axes can be seen as an example of brain-body-environmental coupling, in which upstream and downstream information relevant for the expression of appropriate social behavior are integrated, and therefore can function as a pathway for coordinated convergent adaptive responses to social change (e.g., Oliveira, 2009; Adkins-Regan, 2012). This view follows a soft definition of embodiment, since it still assumes the brain as a central processor that is merely permeable to bodily as well as environmental raw inputs. A more stringent definition of embodiment goes further, by proposing a distributed cognitive system that goes beyond the brain to include the body (therefore spreading the computational load) in an interacting goal-oriented, problem-solving system, that can be exploited by the agent replacing the need for complex internal mental representations (Beer, 2009; Wilson and Golonka, 2013).

**Figure 2:**
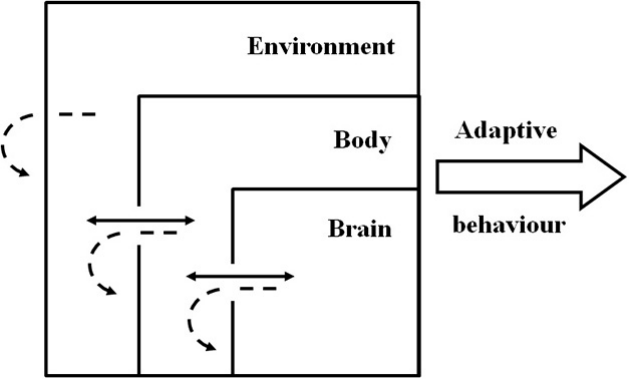
**Schematic representation of the situated-dynamic-embodied framework with adaptive behavior resulting from the emergent characteristics of brain-body-environment coupling and not from singular contribution of the components**. Full arrows represent dynamic processes between the components. Dashed arrows represent the dynamic processes within the components.

But just as the brain is embedded in a body, the body is embedded in an environment. This implies a connection between the behavioral agent and the physical or social environment (situatedness) and therefore the characteristics of the environment and the properties arising from this interaction can also be used by the agent to solve adaptive problems (Beer, 2009; Nolfi, 2011). What arises from this situated-embodied-dynamic framework (Figure 2) is a multi-level complex system in which adaptive behavior and cognition cannot be inferred from any of the elements in isolation as it emerges from the non-linear, dynamical interactions between and within these three foundational elements (Chiel and Beer, 1997; Nolfi, 2011; Williams and Beer, 2013). Examples of this multi-level coupling can be seen in animals, in which adequate locomotion depends not on simple neural commands, but on a multimodal integration of information that must include body and environment feedback (for a review see Dickinson et al., 2000). Also supporting this idea, the body and the morphological characteristics of artificial agents do not simply feed the control center (e.g., brain) with sensory inputs; instead they allow the agent to create or elicit appropriate inputs by actively self-structuring flows of multimodal and temporally specific environmental information into sensorimotor networks, linking information structure from motor activity and information processing in the brain (Lungarella and Sporns, 2005, 2006).

Therefore in embodied agents, a neuromodulatory system, such as the androgen reciprocal model discussed here, must be able to continually guide plasticity, while stabilizing and maintaining previously acquired adaptive structures, and to adapt the agent to variation in behavior, physiology, and external stimuli (Alexander and Sporns, 2002). This definition is compatible with the current hypothesis for the role of androgens on social decision-making mechanisms that has lost the assumptions of causality to focus more on a systems perspective. Empirical evidence for this process can be found in the examples described above (section IV) referring to the effects of T administration, which within a situated-embodied-dynamical framework, can be seen as an experimental manipulation of the information carried by the peripheral signaling of T that is being translated into systemic changes in the brain-body-environment coupling.

Although the neuromodulatory effects of peripheral androgens are well documented, a challenging puzzle arises when one has to account for the dynamics of evolution and the function that peripheral androgens have in this process. If adaptive behavior emerges from brain-body-environment continuous and dynamical interaction, evolution should not select individual components but variations of systemic couplings responsible for the emergent characteristics that originated behavioral efficacy (Beer, 2009). Androgens may play a role in this process by stabilizing the system via pleiotropic effects on neural-dynamics and on relevant body components that could be rapidly enhanced by transient increases in androgens (Oliveira, 2009). Evidence for non-genomic effects on bodily components can be found in the literature (e.g., review by Rahman and Christian, 2007). For example, acute increases of T enhanced 2-deoxyglucose uptake in cultured myotubules within 1 min (Tsai and Sapolsky, 1996) and increased the intracellular concentration of calcium suggesting the existence of a G protein-linked membrane receptor in skeletal muscle cells (Estrada et al., 2003). Also, rapid effects of T on vasorelaxation at micromolar concentrations has been reported in several species (Jones et al., 2004).

In conclusion, the evidence presented here substantiates the need to integrate the proximate mechanisms of behavior with their ecological and evolutionary function as it was proposed by Tinbergen (1963). The apparent paradox of social challenges eliciting increases in peripheral androgen levels at a greater cost (e.g., Wingfield et al., 2001) when brain androgen synthesis is available to the organism, may be better understood by integrating its' action both on neural circuits and on bodily parameters relevant to behavioral performance, influencing the emergent characteristics of the brain-body-environment coupling itself and thus reducing the fitness variability of the expressed phenotypes. Although further research is required to support these claims, previous work by Johnson and Whalen (1988) proposed that in male mice the signaling of gonadal hormones on brain areas is required to regulate and reduce the inter-individual differences in aggressive behavior observed in gonadectomized animals, that are not present in gonadally-intact or castrated mice treated with T. In our view, these experiments can be seen as an example of how the characteristics of the systemic coupling can be skewed into more variable behavioral outputs when body signaling is disrupted, and restored to a finer context dependent response by restituting the signal to congruent levels. This suggests that body signaling is necessary for effective couplings that generate more adaptive patterns of response and this goal could be achieved by narrowing the degrees of freedom for possible fitness outcomes that could be obtained from the multiple combinations of the components involved in the dynamical system. Although most of the research presented here focused on males, this conceptual framework is expectable to also apply to females, at least for humans where recent studies suggest the similar patterns of androgen responsiveness to social competition in both sexes (Jiménez et al., 2012). However, given the possible sex differences in androgen modulation and signaling integration in central systems across different taxa, further research is needed to fully establish this approach in both sexes.

### Conflict of interest statement

The authors declare that the research was conducted in the absence of any commercial or financial relationships that could be construed as a potential conflict of interest.
